# Molecular Insights on Signaling Cascades in Breast Cancer: A Comprehensive Review

**DOI:** 10.3390/cancers17020234

**Published:** 2025-01-13

**Authors:** Venketesh K. Panda, Barnalee Mishra, Samikshya Mahapatra, Biswajit Swain, Diksha Malhotra, Suryendu Saha, Sinjan Khanra, Priyanka Mishra, Sambhunath Majhi, Kavita Kumari, Angitha N. Nath, Swarnali Saha, Sarmistha Jena, Gopal C. Kundu

**Affiliations:** 1 School of Biotechnology, KIIT Deemed to Be University, Bhubaneswar 751024, India; venketeshpanda1998@gmail.com (V.K.P.); barnaleemishra12@gmail.com (B.M.); samikshyamahapatra.ksbt50@gmail.com (S.M.); swainbiswajit555@gmail.com (B.S.); diksha.ksbt@gmail.com (D.M.); suryendu.saha@gmail.com (S.S.); sinjankhanra143@gmail.com (S.K.); priyankamishra1994828@gmail.com (P.M.); smajhi034@gmail.com (S.M.); kavihumangenetics@gmail.com (K.K.); nathankita61@gmail.com (A.N.N.); sahaswarnali2021@gmail.com (S.S.); sarmisthajena99@gmail.com (S.J.); 2 School of Applied Sciences, KIIT Deemed to Be University, Bhubaneswar 751024, India; 3 Kalinga Institute of Medical Sciences (KIMS), KIIT Deemed to Be University, Bhubaneswar 751024, India

**Keywords:** breast cancer, signaling, tumor metabolism, therapeutic hotspot

## Abstract

The complex signaling network within the breast tumor microenvironment is crucial for its growth, metastasis, angiogenesis, therapy escape, stem cell maintenance, and immunomodulation. An array of secretory factors and their receptors activate downstream signaling cascades regulating breast cancer progression and metastasis. Among various signaling pathways, the EGFR, ER, Notch, and Hedgehog signaling pathways have recently been identified as crucial in terms of breast cancer proliferation, survival, differentiation, maintenance of CSCs, and therapy failure. These receptors mediate various downstream signaling pathways such as MAPK, including MEK/ERK signaling pathways that promote common pro-oncogenic signaling, whereas dysregulation of PI3K/Akt, Wnt/β-catenin, and JAK/STAT activates key oncogenic events such as drug resistance, CSC enrichment, and metabolic reprogramming. Additionally, these cascades orchestrate an intricate interplay between stromal cells, immune cells, and tumor cells. Metabolic reprogramming and adaptations contribute to aggressive breast cancer and are unresponsive to therapy. Herein, recent insights into the novel signaling pathways operating within the breast TME that aid in their advancement are emphasized and current developments in practices targeting the breast TME to enhance treatment efficacy are reviewed.

## Simple Summary

Breast cancer is an intricate condition that is caused by aberrant cell signaling regulation induced by the accumulation of genetic and epigenetic alterations. It is well known that various downstream signaling cascades (JAK/STAT, PI3K/Akt, and MAPK) are activated in transformed cells to regulate tumor growth, angiogenesis, metastasis, therapy failure, and stemness. However, the complex signaling networks and their crosstalk is challenged by structural alterations, aberrant gene amplification, and activation of alternative pathways.

## 1. Introduction

Based on GLOBOCAN 2022, approximately 20 million newly diagnosed cancers and 9.7 million cancer-associated mortalities have been reported across the globe. Breast cancer is the most frequent malignancy diagnosed in women, accounting for over 11.6% of new cases and 6.9% of deaths worldwide in 2022 [1]. The frequency of this disease has increased globally over the past two decades, despite a significant rise in survival rates. Hashim et al. reported that adjuvant therapy and mammography screening played a major role in improving survival outcomes [2]. Additionally, current chemotherapy, targeted therapy, and immunotherapeutic regimens have become instrumental in the management of advanced stages of breast cancer.

Breast cancer is composed of tumor, stromal, and immune components within the tumor microenvironment (TME), which make the tumor core conducive to promoting various oncogenic functions. Stromal cells such as cancer-associated fibroblasts (CAFs) and immune cells like tumor-associated macrophages (TAMs), T cells, and mast cells initiate the key oncogenic signaling axis in breast cancer within the TME. Further, recent reports suggest that CAF and TAM subsets also play pivotal role in promoting breast tumor oncogenesis by activation of key signaling cascades [3].

Numerous somatic and genetic abnormalities in breast cancer lead to the aberrant activation of crucial signaling cascades that regulate the differentiation of cells, tumor growth, and metastasis [4]. Genes with low mutational frequency should also be considered, as they are involved in cancer signaling pathways along with highly mutated genes. Additionally, variations in epigenetic regulators including ARID1A, KAT6A, and KMT2C can lead to altered gene expression and have a broad spread [5].

Hanahan and Weinberg identified several hallmarks of cancer, including uncontrolled proliferation, genomic instability, and invasion [6]. Targeting cell cycle-regulatory and proto-oncogenic signaling pathways, including Notch, Wnt, NF-κB, Sonic Hedgehog (SHH), ER, PI3K/Akt/mTOR, and HER2, has become an emerging area in cancer research [7].

In this review, we discuss various fundamental molecular pathways and their crosstalk within the breast TME that result in tumor growth, metastasis, immune escape, and therapy resistance. Moreover, tumor metabolism and CircRNA-, miRNA-, and lncRNA-mediated signaling are also highlighted. Furthermore, various novel therapeutic hotspots and their targeted agents in enhancing breast tumor treatment regimen are discussed here.

## 2. Molecular Classification of Breast Cancer

Human breast cancers are classified by a multifaceted system that provides clinical characteristics, advanced genomic profiling, and histological parameters. Tumors are defined histologically as in situ or invasive carcinomas based on the migration of transformed cells from the lobules or ducts into the stromal component surrounding it [8]. Among all, ductal carcinoma in situ (DCIS), which accounts for about 10–30%, is classified as pre-invasive breast cancer. Invasive carcinomas are categorized based on ducts and lobules such as invasive lobular carcinoma (ILC) and invasive ductal carcinoma (IDC), which account for 10–15% and 60–75% tumor cases, respectively [9]. In addition to their morphological classification, breast cancers are categorized based on ER, progesterone receptor (PR), and HER2 expression. The incidence of these subtypes varies among women based on their receptor status. For instance, about 70% of breast cancers are ER+ve malignancies, whereas HER2+ve tumors can be further classified into HER2+ve ER+ve and HER2+ve ER-ve which account for about 70% and 30%, respectively [9]. Additionally, breast cancer is classified as a luminal A and luminal B subtype. Clinically, luminal A is identified by the expression of ER and/or PR and absence of HER 2 whereas luminal B is characterized by the presence of all three receptors. Luminal B shows a worse prognosis and higher grade of tumors due to the high expression of cell proliferation marker Ki67, while luminal A tumors are clinically low grade [10]. Triple-negative breast cancer (TNBC) (represents 15% among all breast cancers) is the most aggressive and has poor response to therapy due to lack of ER, PR, and HER2 expression. It also has a range of subtypes, the majority of which express CK5 and CK14 cytokeratins as well as EGFR [9]. TNBC cancers typically have an aggressive clinical course associated with a worse chance of survival and a higher grade of diagnosis at a younger age. These cancers predispose sufferers showed early recurrence and metastasis, particularly to the lung, liver, bone, and brain, and account for an abnormally high percentage of breast cancer mortality [11]. Notably, these subtypes and clinical-pathological factors continue to be extremely important when deciding prognosis, therapy choices, and clinical trial design for effective breast cancer management.

## 3. Novel Treatment Regimens in Breast Cancer

### 3.1. Chemotherapy

Chemotherapy is one of the effective treatments for several cancers, including breast cancer [12]. In accordance with mode of interventions, chemotherapeutic drugs are classified as immunologic therapy, anti-metabolites, endocrine therapy, antimitotic agents and DNA alkylating agents [13]. Herceptin (trastuzumab), pertuzumab, and ado-trastuzumab are some of the well-known immunologic agents while methotrexate, capecitabine, 5-fluorouracil, gemcitabine, doxorubicin, palbociclib, ribociclib, and lapatinib (Tykerb) are examples of anti-metabolites; goserelin, megestrol acetate, tamoxifen, and letrozole belong to endocrine therapy; cisplatin, carboplatin, and cyclophosphamide are DNA alkylating agents; and ixabepilone, taxanes, and paclitaxel are antimitotic agents [13]. Cisplatin is shown to impede the migration of the cells in both MDA-MB-231 and MCF-7 via inhibition of early EMT [14]. Furthermore, combination of cisplatin with paclitaxel inhibited both cancer growth at primary site and metastasis without any adverse effects using in vivo breast cancer mice model [14]. Interestingly, the combination therapy of 5-fluorouracil with thymoquinone demonstrated a synergistic effect, thereby significantly enhancing apoptosis and controlling cell growth using triple-negative BT-549 and MDA-MB-231 cells [15]. An international, multicentric, phase III randomized trial, the HERA trial (BIG 1-01), showed a significant improvement in long-term disease-free survival (DFS) for cohorts administered with trastuzumab for one year after chemotherapy as compared with the observation arm or intervention of trastuzumab for two years in patients with HER2+ve early breast cancer [16]. Furthermore, another randomized phase III (PRECIOUS) clinical study evaluated the retreatment efficacy of pertuzumab in HER2+ve metastatic breast cancer patients formerly administered with pertuzumab, trastuzumab, and physician’s choice of chemotherapy (PTC). The results showed better overall survival (OS) of cohorts receiving pertuzumab retreatment in a group that was previously treated with pertuzumab, making it more effective in the third or fourth line of chemotherapy [17]. PD-L1-expressing advanced TNBC patients possessing an overall positive score of 10 or above exhibited a significant increase in OS upon receiving pembrolizumab treatment with chemotherapy when compared with the control (KEYNOTE-355) [18].

### 3.2. Immunotherapy

The advent of the discovery of immune checkpoint inhibitors coupled with several pieces of evidence suggesting the involvement of the immune system in shaping the tumor core and tumor immune microenvironment (TIME) have paved the way for novel and effective therapeutic strategies for breast cancer, including TNBCs [19]. The IMpassion130 trial demonstrated the efficacy of treatment with atezolizumab and nab-paclitaxel in extending progression-free survival (PFS) in patients with metastatic TNBC [20]. However, owing to the lack of clinical benefits, the FDA revoked the approval for atezolizumab in breast cancer treatment [21]. Similarly, evaluation showed that the combination of atezolizumab with paclitaxel failed to improve the PFS and OS in metastatic TNBC patients (IMpassion131) [22]. A phase Ib study involving hormone receptor-positive (HR+ve) and HER2-ve metastatic breast cancer patients receiving treatment of abemaciclib plus pembrolizumab with/without anastrozole evaluated the efficacy and safety of the combination [23]. Even though this combination of drugs showcased anti-tumor activity, it showed adverse side effects including pneumonitis led to the termination of further evaluation [22]. However, the combination of palbociclib, letrozole, and pembrolizumab in patients with HR+ve and HER2-ve stage IV metastatic breast cancer was well tolerated and demonstrated a PFS of 25.2 months [24]. The combination of pembrolizumab with trastuzumab demonstrated anti-tumor activity in patients with PD-L1+ve, metastatic, trastuzumab-resistant, HER2+ve breast cancer [25]. However, the atezolizumab plus trastuzumab emtansine combination was associated with an increase in adverse side effects rather than any significant improvement in PFS of PD-L1+ve and HER2+ve advanced breast cancer patients [26].

### 3.3. Targeted Therapy

Breast cancer heterogeneity demands that treatment regimens and therapies be precise and well targeted to a specific grade, subtype, and stage [27]. The addition of exemestane, an aromatase inhibitor to everolimus, an mTOR inhibitor, demonstrated tolerability, safety, and efficacy in HR+ve and HER2-ve metastatic breast cancer patients [28]. In a phase II clinical study involving postmenopausal women with HR+ve and HER2-ve metastatic breast cancer, a combination of tamoxifen with everolimus significantly increased OS and clinical benefit as compared with the control cohorts [29]. In HR+ve, HER2-ve, and PIK3CA-mutated breast cancer patients with previous endocrine treatment, the combination of α-specific PI3K inhibitor, alpelisib (BYL719), with fulvestrant demonstrated longer PFS [30]. In addition, combination of paclitaxel with pictilisib (GDC-0941)—a PI3K inhibitor with/without trastuzumab or bevacizumab—or letrozole showcased anti-tumor activity along with fewer adverse effects [31]. In HER2-ve metastatic gBRCA-associated breast cancer patients, combination of veliparib with carboplatin and paclitaxel yielded a significant improvement in PFS without altering the toxicity profile of carboplatin and paclitaxel [32]. Olaparib monotherapy in HER2-ve metastatic breast cancer patients bearing BRCA mutation showed an increase in PFS by 2.8 months and reduction in death or disease progression by 42% when compared with the standard therapy [33]. Recent FDA-approved drugs for breast cancer treatment are summarized in Table 1.

## 4. Oncogenic Signaling in Breast Cancer

**Figure 1:**
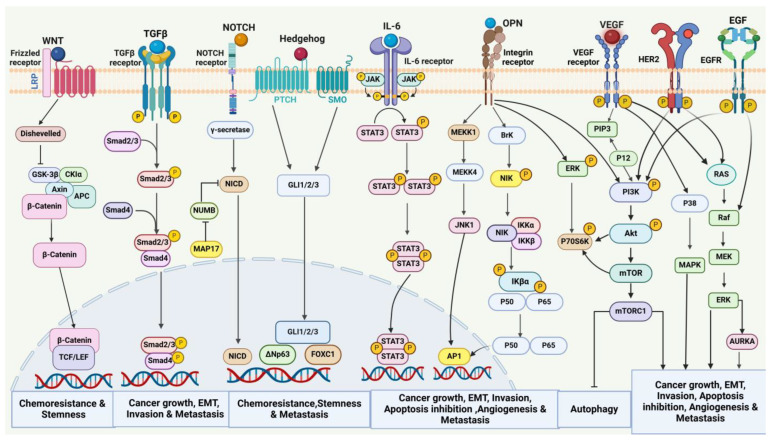
Various cell signaling cascades involved in breast cancer. The major signaling events, including Wnt/β-catenin, TGF-β, Notch, MAPK, Hedgehog, JAK/STAT, PI3K/Akt/mTOR, and NF-κB pathways, and their involvement in the regulation of tumor progression, survival, and metastasis. Wnt/β-catenin pathway is responsible for enhancing the stemness and promoting chemoresistance. TGF-β-mediated signaling is associated with the growth, invasion, and metastasis of breast cancer. Notch pathway as well as Hedgehog signaling induce chemoresistance and metastasis. JAK/STAT and NF-κB signaling cascades modulate breast cancer growth, invasion, metastasis, and angiogenesis. VEGFR-, HER2-, and EGFR-mediated PI3K/Akt/mTOR, MAPK, and ERK pathways have a key role in the regulation of breast tumor growth, metastasis, angiogenesis, and apoptosis.

Cell signal transduction is crucial in breast cancer development and progression. Alterations to various cell signaling cascades regulate tumor cell proliferation and survival. Dysregulation of signaling pathways, such as TGF-β, Wnt, Hedgehog, Notch, IL-6, Integrins, VEGF, HER2, EGFR, PI3K/Akt, and MAPK, leads to breast cancer cell proliferation and metastasis, as depicted in Figure 1.

### 4.1. Involvement of EMT in Breast Cancer Invasion and Metastasis

Epithelial-to-mesenchymal transition (EMT) is a process by which epithelial cells gain mesenchymal properties, crucial for cancer propagation and metastasis. The mesenchymal-to-epithelial transition (MET) allows for mesenchymal cells to re-differentiate into epithelial cells [34]. This transition plays a role in wound healing, fibrosis, and tumor progression, but cancer cells during EMT gain migratory abilities crucial for malignancy. They can invade other tissues through blood or lymph vessels, where they undergo MET, converting back into epithelial cells. The stem cell characteristics of EMT-derived tumor cells make them resistant to therapy, leading to the development of therapies targeting EMT for metastatic patients [35].

Transcription factors (TFs) regulate gene expression by binding to chromatin, playing a crucial role in cancer advancement and metastasis. EMT-TFs can inhibit E-cadherin, a protein facilitating cancer cell invasion [36]. Loss of E-cadherin is linked to TGF-β upregulation, reactive oxygen species, and changes in apoptotic signaling pathways [37]. Inhibiting E-cadherin increases mesenchymal cell markers like N-cadherin and vimentin. TWIST1, SLUG, SNAIL, ZEB1, ZEB2, and FOX families inhibit E-cadherin, impacting cancer initiation, progression, invasion, metastasis, and treatment resistance [38]. EMT-TF overexpression in breast cancer predicts metastasis and promotes cancer growth. TWIST1 enhances E-cadherin promoter hypermethylation and hypoacetylation, driving metastasis while knockdown of TWIST1 inhibits metastasis in breast cancer [39]. Further, SNAIL inhibits E-cadherin expression via vimentin upregulation, facilitating EMT [40]. SNAIL interacts with Suv39H1, contributing to breast cancer metastasis through epigenetic processes [41]. The ZEB family suppresses E-cadherin expression, promoting vimentin and N-cadherin expression. ZEB1 acts as an interactive partner with AP-1 to form a transactivation complex with YAP and stimulate aggressive claudin-low subtype of breast cancer [42]. These findings highlight the significance of EMT-TFs in cancer progression and underscore their potential as therapeutic targets for treating metastatic breast cancer.

Current research depicts that cells with stem cell properties are indispensable for the growth and spread of tumors [43,44]. While the exact origin of cancer stem cells (CSCs) remains elusive, biomarkers for breast cancer stem cells (BCSCs) have been identified, such as CD44+ve/CD24−ve/low cells that exhibit aggressive behavior and drug resistance [45]. Moreover, high levels of aldehyde dehydrogenase 1 (ALDH1) activity have been associated with poor breast cancer clinical outcomes [46,47]. These BCSCs show enhanced self-renewal and tumor initiation abilities and express markers like TWIST1 and FOXF2 [48]. Studies have shown that SLUG increases cytoplasmic β-catenin stability by inducing certain inflammatory factors such as IL-8 and TNF-α, while TWIST1 regulates CD24 expression, promoting stem cell characteristics in breast cancer [49,50]. Transcription factors like Lin28B and CCAAT/enhancer binding protein δ (C/EBPδ) also play important roles in the metastatic niche formation, whereas Lin28B is involved in lung metastases of breast cancer [51,52]. Additionally, IL-6 supports CSC survival and treatment resistance, while C/EBPδ contributes to CSC maintenance and metastasis through its linking with hypoxia-inducible factor-1 (HIF-1) and IL-6 [53]. Overall, understanding the role of CSCs and related transcription factors is crucial in developing targeted therapies for breast cancer metastasis.

### 4.2. Angiogenesis

Angiogenesis is a process of the formation of new blood vessels induced by various pro-angiogenic factors and is predominantly involved in embryonic development and wound healing in physiological conditions. In pathophysiological conditions like cancer, blood vessels carry and supply essential nutrients for promoting tumor growth, survival, proliferation, and metastasis. Further, this process is governed by pro-angiogenic and anti-angiogenic factors, where vascular endothelial growth factor (VEGF) is a pivotal player while other factors coordinate to build an environment to foster a vascular network for tumor progression and development.

#### 4.2.1. VEGF-Dependent Angiogenesis

The VEGF family consists of seven components: placental growth factor (PlGF), VEGF-A, VEGF-B, VEGF-C, VEGF-D, VEGF-E, and snake venom vascular endothelial growth factor (svVEGF) [54]. VEGF-A is a key secretory factor that modulates cell mitosis, vascular permeability, and human endothelial functions [55]. It exists in various isoforms, including VEGF121, VEGF165, VEGF189, VEGF206, VEGF111, and VEGF145, that vary in molecular size and tissue distribution [56]. It also contributes to cell homeostasis, invasion via autocrine or paracrine mechanisms, and hematopoietic stem cell as well as tumor cell survival. Furthermore, VEGF-A is an essential angiogenesis regulator, having critical roles in tumor growth, proliferation, angiogenesis, invasion, metastasis, and treatment resistance [57]. VEGF induces angiogenesis by interacting with its primary receptors: VEGFR-1/Flt-1, VEGFR-2/KDR, and VEGFR-3/Flt-4 [58]. Zhu et al. have demonstrated that IGF-1 enhances the expression of VEGF-C via the PI3K/Akt and MAPK/ERK1/2 signaling pathways in MDA-MB-231 breast cancer cells. Additionally, they have proposed that the IGF-MAPK/ERK1/2-VEGF-C and IGF-1-PI3K/Akt-VEGF-C signaling pathways are crucial for lymph angiogenesis in breast cancer [59]. VEGFR-2 is a key mediator of angiogenesis. VEGFR-2-axis induces cell proliferation through MAPK/ERK signaling cascade as well as cell survival, migration, and vascular permeability via the FAK/PI3K/Akt signaling [60].

Breast cancer frequently exhibits overexpression and activation of HIFs, which are key regulators for cells to adjust to low cellular oxygen levels. In response to hypoxia, HIFs alter the primary transcriptional response of downstream pathways and target genes involved in glycolysis, angiogenesis, and metastasis [61,62]. Hypoxic conditions in tumors can promote growth and survival by inducing angiogenesis in response to osteopontin (OPN) and VEGF [63].

Moreover, pathways such as ILK/Akt/NF-κB/p65 axis or FOXO3a dependent MelCAM upregulation controls angiogenesis in breast cancer [63,64]. Breast tumor angiogenesis can be mediated by autocrine, juxtacrine, and paracrine mechanisms via the OPN/NRP-1/Brk/NF-κB/ATF-4 signaling pathway [65]. Von Willebrand Factor (vWF), a pro-angiogenic factor, promotes angiogenesis by triggering VEGF-A via PI3K/Akt/miR-205-5p pathway in breast cancer cells [66]. Aurora kinase A (AURKA) found in spindle microtubules and centrosomes aids cell division in normal conditions. In breast cancer, elevated AURKA levels lead to VEGF-dependent angiogenesis via the ERK pathway [67]. Moreover, deciphering these pathways may aid in the development of targeted therapies for breast cancer treatment.

#### 4.2.2. VEGF-Independent Angiogenesis

Studies on tumor vascularity mainly focus on VEGF-dependent angiogenic therapy; however, it is also crucial to investigate VEGF-independent tumor angiogenesis. Wan et al. have reported that FOSL2 activates Wnt5a transcriptional signaling and thus promotes angiogenesis in cancer-associated fibroblasts (CAFs) [68]. CAFs play a major role in tumor growth and metastasis. This facilitates the pre-metastatic niche through lncRNA SNHG5 by enhancing the vascular permeability in breast cancer [69]. Pearson’s correlation analysis suggested a link between CD31/34 and lncRNA NR2F1-AS1 and further proved that lncRNA NR2F1-AS1 activates IGF-1/IGF-1R/ERK pathways that promotes angiogenesis and metastasis using in vitro and in vivo breast cancer models [70]. Cao et al. investigated decylubiquinone (DUb), a coenzyme that suppresses angiogenesis by targeting the ROS/p53/BAI1 signaling pathway [71].

Integrins, a type of cell adhesion receptor, play important roles in cell–cell and cell–matrix interaction in normal cells whereas they dysregulate in tumor cells. Abnormal conditions such as hypoxia and glycosylation alter the function of several integrins, resulting in the activation of key signaling cascades upon interaction with pro-tumor secretory factors such as VEGF, FGF, and PDGFR2; therefore, integrins are a novel target for angiogenesis in cancer therapy [54]. ATN-161 (Ac-PHSCN-NH2) consists of five amino acids, commonly binds to integrin (α5β1), and thereby suppresses the angiogenesis and bone metastasis in breast cancer [72]. Excessive neovascularization or vascular mimicry in breast tumor-initiating cells (BTIC) increase the occurrence of TNBC development. ASB10 (estrogen receptor ER-α trans-activated E3 ligase) ubiquitylates TEM8 (tumor endothelial marker 8); thus, the deficiency of ASB10 resulted in high accumulation of TEM8, leading to enhancements in vascular mimicry (VM). A high level of TEM8 enhances the active RhoC level, which promotes ROCK1-dependent SMAD5 phosphorylation, further enhancing the stemness and VM in breast cancer cells [73]. Vimentin, in its extracellular region, mimics as VEGF, acts as a stimulator to upregulate VEGFR-2 and PD-L1 receptors, and promotes angiogenesis [74].

### 4.3. Cancer Stem Cells

The multipotent capacity of BCSCs has been implicated in enhancing several hallmarks of cancer within the TME. In the breast TME, mammary stem cells (MaSCs) were primarily found in the outer basal areas and consisted of diverse subsets of BCSCs with distinct molecular signatures [75]. Similarly, other conventional stem cell markers such as CD133-, CD44-, CD49f-, and ALDH-expressing stem cells regulate various signaling networks, including the Notch, Hedgehog, and Wnt pathways, for maintenance and proliferation in breast cancer.

BCSCs exhibit oncogenic features such as tumor growth, metastasis, tumor angiogenesis, immunomodulation, and treatment resistance. For instance, BCSC secretome analysis revealed that MIF activates Wnt/β-catenin signaling to upregulate the glycolytic enzyme aldolase C through c-MYC transcription [75]. Further, Xie et al. reported that overexpression of XB130 in various malignancies is linked to poor survival and increased EMT, a necessary cellular phase for BCSC induction, and accelerated tumor initiation through the Wnt/β-catenin signaling pathway [76]. In addition, the co-transcription factor Limb–Bud-Heart (LBH) targets genes involved in Wnt/β-catenin signaling and activates stem cell transcription resulting in breast cancer metastasis [77]. A previous study found that blocking cadherin 11 downregulates Wnt/β-catenin signaling pathway, resulting in a decrease in the cancer stem-like phenotype [78]. Overexpression of CCL2 enhances stemness and macrophage polarization in breast cancer via STAT3 and Notch-1 signaling cascade, whereas silencing it increases tumor necrosis and autophagy, resulting in reduced CSC population and macrophage recruitment. The same impact was not found upon neutralization of CCL2 [79]. Further, binding of KK-LC-1 to FAT1 increases its ubiquitination and degradation. This disrupts the Hippo pathway, resulting in nuclear translocation of YAP1 and ALDH1A1 transcription. Z839878730 (Z8) is a small-molecule inhibitor that can interfere with KK-LC-1 and FAT1 interaction, resulting in reduced stemness in TNBC [80]. SDF-1/CXCL12 stimulates the NF-κB pathway, resulting in high ALDH activity and upregulation of OCT-4, Nanog, and SOX2, increasing the BCSC population, leading to the metastasis of MCF-7 cells [81]. Shan et al. discovered that overexpression of CXCL12 induces MCF-7 cells to develop BCSC phenotype through the Wnt/β-catenin pathway, leading to enhanced metastasis [82]. Polymorphonuclear myeloid-derived suppressor cells regulated by CCL20 derived from cancer cells increased stemness via the CXCL2-CXCR2 pathway [83]. Moreover, the lncRNA LINC00115 enhances chemoresistant stem-like cells, enriches stemness, and promotes metastasis via the SETDB1/PLK3/HIF1α axis in breast cancer [84].

Moreover, the link between EMT and CSCs is well established. For instance, breast cancer cells’ stemness and tumorigenicity are linked to the attainment of a hybrid or partial E/M phenotype. Liu et al. have shown the link between the heterogeneity in BCSCs and EMT. The CD24−ve/CD44+ve/ALDH+ve subpopulation with intermediate EMT state possess the maximum potential for stem cell enrichment and carcinogenesis [85]. Pasani et al. demonstrated that enrichment of intermediate E/M phenotypes promotes stemness, which is more likely to occur in these phenotypes than in “pure” E/M phenotypes using a mechanism based mathematical models [86]. Brown et al. found that CBFβ stabilized and maintained metastatic potential in three single-cell clones in the intermediate E/M stage. The study found that CBFβ had a higher prognostic value for survival outcomes than EMT score alone [87].

Tumor CSCs have diverse metabolic states and can adapt to different conditions. Somatic stem cells, embryonic stem cells, and induced pluripotent stem cells have all exhibited elevated glycolysis activity to preserve their stem cell characteristics. Luo et al. demonstrated the transition of epithelial to mesenchymal state depends on the modulation of redox metabolism [88]. By lowering ROS and ferroptosis, NRF2 mirrored ZMYND8 and improved breast cancer stemness and tumor formation. The loss of NRF2 abolished ZMYND8 effects on antioxidant genes and ROS in mammospheres. Interestingly, NRF2 directly affected ZMYND8 expression in mammospheres [88].

### 4.4. Tumor Metabolism

**Figure 2:**
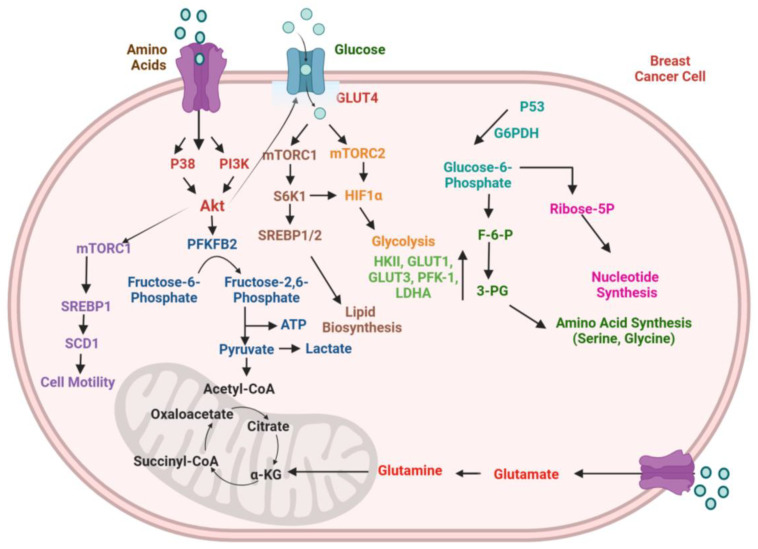
The key signaling and transcription factors associated with energy metabolism networks that serve as the foundation for metabolic reprogramming in the progression of breast cancer.

Breast cancer, like other malignancies, has been linked with metabolic reprogramming. Breast cancer manifests improved glycolysis, glutamate metabolism, pentose phosphate pathway (PPP), tricarboxylic acid (TCA) cycle, and lipid metabolism, across energy metabolic pathways. These pathways are rewired to promote breast cancer growth, proliferation, and migration [89]. HIF, TP53, c-Myc, extracellular acidification, and interactions with immune cells, CAFs, and adipocytes are a few of the intrinsic factors that have been documented to be mutated or inactivated in breast cancer cells. The underlying cause for metabolic reprogramming in the progression of breast cancer is the abnormal expression of several signaling and transcription factors associated with energy metabolism networks, as shown in Figure 2 [90].

#### 4.4.1. Glucose Metabolism

The connection between breast cancer and aerobic glycolysis has been closely studied recently [91]. The key molecular signaling involved in the regulation of aerobic glycolysis in breast cancer are the mTOR, PI3K/Akt, and AMPK pathways. PI3K/Akt modulates the phosphorylation and activation of phosphofructokinase-2 (PFK-2) [92,93]. GLUT-1 overexpression results from activation of the PI3K/Akt signaling pathway, and this leads to its translocation from the cytoplasm to the plasma membrane [94]. Beta-estradiol (E2) activates Akt, which causes GLUT-4 to move to the plasma membrane and increases the uptake of glucose in the MCF-7 cell line [95]. PIK3CA and Akt1 gene mutations are frequently observed in breast cancer, with PIK3CA mutations primarily being detected in HER2+ve and ER+ve breast cancer [96,97]. Estrogen causes overexpression of c-myc, and approximately 80% of breast tumors are ER+ve [98,99]. Glycolysis-related enzymes like phosphofructokinase (PFK) are expressed more when HIF-1α expression is promoted and the switch to glycolysis from OXPHOS is stimulated by mTORC1 whereas mTORC2 promotes glycolysis by inducing Akt and GLUT-1-associated glucose absorption [100,101,102]. Moreover, HIF-1α boosts the expression of molecules linked to glycolysis, such as HKII, PFK-1, GLUT-1, GLUT-3, and lactate dehydrogenase (LDH) A [103]. However, p53 gene alterations are seen in the majority of malignancies, including breast cancer [104]. p53 inhibits the expression of GLUT-1, GLUT-3, GLUT-4, and phosphoglycerate mutase (PGM); thus, p53 mutation causes enhanced glycolysis in breast cancer [104]. Furthermore, p53 controls glycolysis and GLUT via mTOR and AMPK, and the PI3K/Akt/mTOR pathway stimulates the production of glycolytic enzymes and GLUT [103,105].

Lymphocytes in the TME have been linked to better prognosis and survival in various cancers and are implicated in anti-tumor immune responses. Immune cell failure, tumor cell proliferation and invasion are facilitated by abnormal glucose metabolism in the TME. The regulation of T cell function is somehow accomplished through metabolic modulation, and glucose metabolism plays a crucial role in the proliferation, activation, and function of T cells. The primary Ca^2+^ influx channel in T cells, for instance, is store-operated Ca^2+^ entry (SOCE). By controlling T cell glucose metabolism, the SOCE/calcineurin/NFAT pathway can regulate T cell development, proliferation, and function. Evidence suggests that an acidic TME, as well as elevated lactic acid, can have a major impact on macrophages [106]. For example, reduced pH in the TME can affect macrophage phenotype and functionality independently [107]. Lactic acid, in particular, produced by cancer cells, plays an important signaling role in the TME by inducing M2 polarization [108]. Furthermore, when M1 and M2 macrophages were incubated at pH 7.4 or 6.8, M2 macrophages demonstrated greater survivability and fitness in the lower pH than their M1 counterparts [109]. Pro-inflammatory M1 markers (e.g., iNOS, MCP1, IL-6) were similarly reduced at acidic pH, but M2 markers (e.g., MRC1, arginase 1 (Arg1), chitinase-3-like protein) were increased [109].

#### 4.4.2. Lipid Metabolism

The reprogramming of lipid metabolism in cancer has been attributed to cancer-associated signaling pathway activation and interactions within the TME [110]. Lipid metabolism holds significance in tumor immunogenicity by regulating the activities of non-cancerous cells in the TME, particularly immune-associated cells [111].

In breast cancer, a decreased level of GPX4, a critical factor that regulates the oxidation of glutathione, prevents lipid peroxide formation as well as ferroptosis [112]. It also increases the production of pentaspanin protein prominin-2, which enables the formation of ferritin-containing exosomes. Both breast cancer cells and mammary epithelial cells become resistant to ferroptosis as a result of this mechanism [113]. Interestingly, GPX4 expression has shown an exceptional prognostic potential in breast cancer neoadjuvant therapy, and a high level of GPX4 is significantly correlated with metastasis-free survival [114]. Stearoyl–CoA desaturase (SCD1) is an important modulator of ferroptosis. PI3K/Akt/mTOR pathway activation results in enhanced synthesis of monounsaturated fatty acids, which leads to the increase in cell motility through downstream sterol regulatory element-binding protein 1 (SREBP1)-mediated upregulation of SCD1. This mechanism safeguards breast cancer cells from ferroptosis induced by ROS [115]. Sphingodylcholine (SPC) is a lipid mediator found in the blood that controls both apoptosis as well as autophagy. Interestingly, autophagy negatively regulates SPC-mediated apoptosis in TNBC cell lines. SPC promotes apoptosis by blocking c-JNK signal transduction and induces autophagy via the Akt/p38 signaling pathway [116].

EMT in breast cancer is fueled by various mechanisms that are influenced by the numerous lipid metabolic pathways and their intermediates. SREBP1 recruits the Snail/HDAC1/2 complex to decrease the expression of E-cadherin, and miR-18a-5p has been recognized as a possible regulator of SREBP1. SREBP1 upregulation and miR-18a-5p inhibition both notably increase the likelihood of breast cancer metastasis [117]. Sphingomyelin homeostasis is majorly regulated by the overexpression of sphingomyelin synthase 2 (SMS2) in breast cancer. By elevating TGF-β1 activity, it activates the TGF-β/Smad signaling pathway, which promotes EMT and increases breast cancer cell invasion and metastasis [118]. Targeting altered lipid metabolism processes may have potential as an anticancer treatment.

#### 4.4.3. Immunometabolism

The abnormal metabolism in tumor cells leads to an acidic, hypoxic environment. In the aberrant metabolic microenvironment, the immune system experiences nutrient deprivation and metabolic alterations that impact their activation [119]. For example, CD28 promotes glucose absorption and glycolysis through the PI3K/Akt signaling pathway, enabling T cells to maintain their activity [105]. When T cells receive PD-1 signals, they utilize enhanced FAO to produce energy and lipolysis by elevating the expression of ATGL and CPT1A. Also, CTLA-4 may inhibit glycolysis without increasing FAO [120]. Recent investigations show that PD-1 signaling plays a role in the FAO in T cells [121]. Clinically employed checkpoint blockade antibodies against CTLA-4, PD-1, and PD-L1 restore glucose in the TME, allowing for T cell glycolysis and generation of IFN-γ. PD-L1 is a crucial regulator of tumor glucose metabolism, as demonstrated by studies indicating that inhibition of PD-L1 disrupts glycolysis by suppressing mTOR activity and decreasing glycolytic enzyme expression [122]. PD-L1 depletion can lower the rate of glycolysis by suppressing the expression of glycolytic enzymes and mTOR activity, indicating that PD-L1 may be essential for the uptake of glucose in cancer cells [122,123].

### 4.5. Autophagy

The essential cellular function that includes the disintegration and recycling of intracellular components is autophagy [124,125]. Based on the method by which protein enters lysosomes, autophagy may be categorized into four groups—microautophagy, macroautophagy, selective autophagy, and chaperone-mediated autophagy [126]. While cells are under stressful conditions, ULK1 is directly or indirectly activated, which phosphorylates Beclin-1, and ATGs, in turn, permits the assembly of molecular complexes and the initiation of phagophore formation [127,128]. Lower Beclin-1 expression is observed in almost 70% of breast cancer specimens [129]. As a result of Beclin-1 gene overexpression in breast cancer, MCF7 cells have been shown to increase autophagic activity; thereby, cell growth and tumor formation are reduced using in vivo breast cancer models [130]. The next stage of the autophagic pathway is the elongation stage, which involves a variety of proteins like ATG12, ATG10, ATG16, ATG7, ATG3, ATG5, LC3, and others [131]. During the maturation stage, autophagosomal membrane degradation is started by SQSTM1(p62), which interacts directly with LC3 and ubiquitination-related proteins [132]. 

Numerous studies have revealed an important connection between autophagy and the activation of different signaling pathways either directly or indirectly in breast cancer. One of the most extensively examined pathways associated with autophagy is the Akt-mTOR signaling pathway. Knockdown of OPN suppresses the PI3K/Akt/mTOR signaling pathway and increases autophagy via regulating the expression of αvβ3 integrin [133]. Eugenol induced apoptosis and autophagy by inhibition of the PI3K/Akt/FOXO3a signaling pathway in breast cancer cells [134]. Chaga mushroom extract (CME) treatment enhances LC3 expression and AMPK phosphorylation but decreases S6, S6K1, and mTOR phosphorylation. These findings imply that CME promotes autophagy by inhibiting the AMPK pathway [135]. The NF-κB signaling pathway is well connected with the autophagic pathway in breast cancer. It has been reported that miR-1910-3p, which targets MTMR3 and activates the NF-κB pathway within exosomes, increases proliferation, autophagy, and metastasis in breast cancer [136]. Through the JNK1-Bcl2 signaling pathway, BMP4 has been shown to cause protective autophagy and apoptosis in breast cancer [137]. Additionally, autophagy has the ability to influence the activation of some tumor signaling pathways such as the knockdown of ATG4A, which suppresses Wnt pathway-related protein expressions [138]. Thus, targeting autophagic molecular cascades could open new dimensions in the treatment of breast cancer.

## 5. Stromal Cell-Mediated Signaling in Breast Cancer

**Figure 3:**
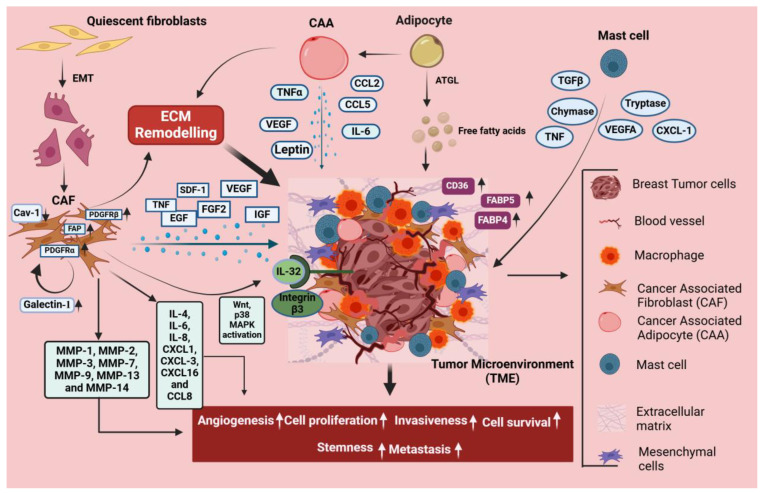
Culmination of signaling pathways in the tumor stroma microenvironment involving cancer-associated fibroblasts (CAFs), adipocytes, and mast cells. CAFs release growth factors like TNF, SDF-1, VEGF, EGF, FGF2, and IGF, resulting in breast cancer progression. Secretion of several MMPs by CAFs such as MMP1, MMP2, MMP3, MMP7, MMP9, MMP13, and MMP14 are responsible for breast cancer advancement. Cytokines and chemokines released from CAFs include IL4, IL6, IL8, CXCL1, CXCL3, CXCL16, and CXCL8, which enhance breast cancer cell motility and aggressiveness. CAAs secrete inflammatory adipokines such as TNFα, leptin, CCL2, CCL5, and IL6, which modulate breast cancer progression. Mast cells contribute to angiogenesis in breast cancer by releasing growth factors such as VEGF, FGF2, and PDGF, along with proteases such as tryptases and chymases.

Breast cancer growth is strongly governed by stromal cells, such as mast cells, adipocytes, tumor-associated macrophages (TAMs), and CAFs, which facilitates invasion, angiogenesis, and immune suppression by promoting tumor growth through TGF-β, VEGF, and cytokine signaling, as illustrated in Figure 3. Mast cells provide pro-inflammatory signals that promote tumor growth, whereas adipocytes provide energy and secrete substances that improve cancer cell survival.

### 5.1. CAFs

The TME comprises numerous cell types that participate in the enhancement of tumorigenicity and modulate cancer aggressiveness. Breast cancer consists of neoplastic cells and is also witnessed with significant alterations in the TME. Several components in the breast TME suppresses immune cells, secrete soluble factors, and alter functionality of the extracellular matrix, which together act to promote breast cancer progression, anti-tumor immunity, and metastasis [139].

CAFs comprise the major part of the tumor stroma and affect the TME by aiding in cancer proliferation, angiogenesis, invasion, and metastasis [140]. Studies suggest that CAFs contribute greatly to regulating and shaping the tumor metabolism through dysregulation of several metabolic pathways, including amino acid, glucose, and lipid metabolism [141]. Activation of CAFs is an irreversible process, and almost 80% of the stromal fibroblasts in breast cancer attain an altered phenotype manifested by the secretion of elevated levels of growth factors, cytokines, and MMPs [142]. Upon activation, these CAFs interact with the neighboring tumor cells continuously, which leads to breast cancer progression by releasing growth factors such as fibroblast growth factor 2 (FGF 2), insulin-like growth factor (IGF), CXCL12 or stromal-derived growth factor (SDF-1), EGF, TNF, VEGF2, as well as cytokines and chemokines such as CCL8, CXCL16, IL-4, IL-6, IL-8, CXCL1, and CXCL3, which increase breast cancer cells’ motility [143]. The metabolic reprogramming of CAFs is initiated in the TME, involving events like the Warburg effect, shifts in Kreb cycle metabolites, and an increased rate of oxidative phosphorylation that serves as the energy source for breast tumor growth and invasion [144]. Luga et al. reported the production of exosomes by CAFs, which boost the ability of breast cancer cells to metastasize by activation of Wnt signaling [145]. Studies have shown that CAFs elevate cancer cell proliferation, invasion, and metastasis by secreting MMPs such as MMP1, MMP2, MMP3, MMP7, MMP9, MMP13, and MMP14 using in vivo and in vitro breast cancer models [143]. It has also been observed that galectin-1, highly expressed in CAFs, regulates the activation of CAFs and leads to upregulation of MMP-9, which further promotes metastasis in MDA-MB-231 cells [146]. Moreover, in breast cancer, the loss of caveolin-1 in stromal fibroblasts is observed to be an independent predictor of nodal metastasis, tumor recurrence, and poor clinical prognosis, whereas elevated levels of caveolin-1 are associated with increased survival [147,148]. Wen et al. demonstrated that CAFs produce IL-32, which further binds to integrin β3, thereby activating p38MAPK signaling, thus enhancing the expressions of fibronectin, vimentin, and N-cadherin in the breast cancer cells [149]. Tchou et al. showed that CAFs derived from HER2+ve breast cancer prominently augmented invasive properties involving pathways concerned with migration of cancer cells, and that these cells confer more invasiveness than TNBC and ER+ve cancers [150]. Choi et al. have demonstrated that CAFs enhance transmigration of breast cancer cells via the blood–brain barrier by boosting the expression of αvβ1 and α5β1 integrins [151]. Moreover, enhanced levels of all CAF-related proteins such as PDGFRα, PDGFRβ, and FAP are reportedly associated with cancer invasiveness and more likely to be found in HER2 subtypes than in TNBC [152]. Hence, targeting the tumor–stroma interaction serves as a promising ground for the advancement of therapeutics, potentially augmenting the existing treatments and preventive options for breast cancer [153].

### 5.2. Adipocytes

Adipocytes in the TME possess a multifaceted role in triggering the interaction between the stromal compartment and breast tumor core. The role of adipocytes in breast cancer growth is quite evident, considering that adipocytes have metabolic and endocrine functions along with the storage of fatty acids [154]. It has been observed that upon prolonged interaction with tumor cells, almost all lipid droplets disappear from adipocytes, resulting in remarkable changes in morphology, more likely towards a fibroblast-like shape [155]. The loss of lipid content from the adipocytes in tumor stroma indicates that free fatty acids are released from these cells and transferred to the tumor cells, and this transfer of fatty acids from adipocytes to tumor cells via the adipose triglyceride lipase (ATGL)-dependent lipolysis pathway boosts tumor growth in vivo and in vitro, supporting the overexpression of pro-inflammatory cytokines and proteases, which leads to the activation of cancer-associated adipocytes (CAA) [156,157]. The regulatory mechanisms of CAA in breast cancer are complex, including inflammatory adipokine secretions such as IL-1β, CCL2, TNFα, CCL5, and leptin, as well as IL-6 metabolic reprogramming by altering the metabolism of macronutrients and remodeling of the extracellular matrix [158,159]. Earlier reports revealed that fatty acids from adipocytes cause breast cancer cells to undergo metabolic reprogramming [156]. This involves an increase in mitochondrial fatty acid oxidation, uncoupled with an increase in anaerobic glycolysis or ATP synthesis [156]. It has further been observed that adipocyte-secreted free fatty acids result in the activation of AMPK in breast cancer cells, which is a key autophagy regulator [156]. Zaoui et al. have demonstrated that adipocyte-conditioned media stimulates CD36 expression in breast cancer cells, and CD36 activity contributes to adipocyte-induced cancer cell migration and invasion [160]. Obese people with breast cancer have been found to have higher levels of FABP4, which is associated with aggressiveness and stemness in breast cancer [161]. Higher levels of FABP5 in breast cancer cells that interact with adipocytes are associated with increased cancer aggressiveness [162].

### 5.3. Mast Cells

Mast cells play a significant role in both autoimmune and chronic inflammation of illnesses, as well as playing a role in the growth of tumors. It is widely recognized that mast cells induce neovascularization and angiogenesis by secreting VEGF, FGF-2, PDGF, and IL-6 to the tumor stroma in addition to nonclassical pro-angiogenic drivers such as proteases, particularly tryptases and chymases [163]. Mast cells are known to localize at the margins of tumor stroma, commonly around the vessels [164]. Mast cells accumulate in the TME by the release of chemoattractants by tumor cells, including SCF or CCL15 [163]. The resting state of mast cells is generally less abundant in breast tumors compared to normal tissues, whereas the activated phenotype of mast cells is seen in increased numbers in breast tumors [165]. Targeting mast cells represents a potential strategy for therapeutics because they represent the key components of immune tumor infiltration and play a crucial role in angiogenesis [166].

### 5.4. TAMs

**Figure 4:**
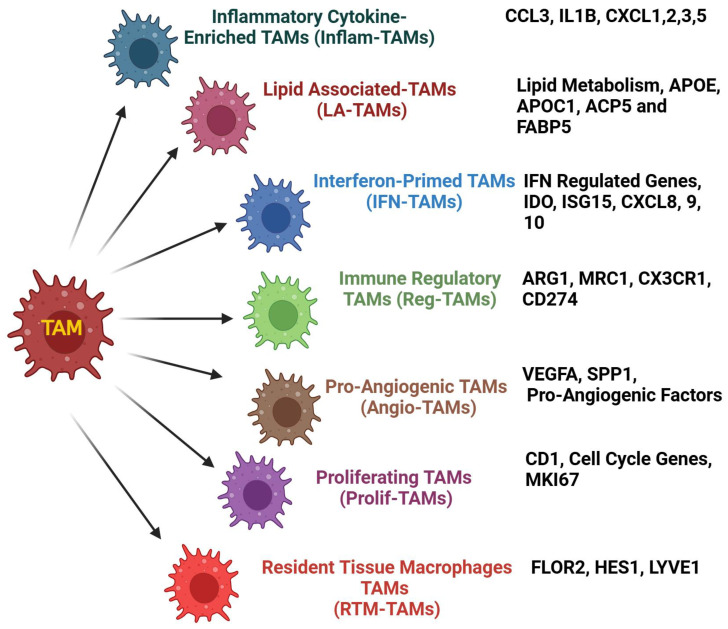
Schematic representation of various TAM subsets based on their molecular signatures and secretory factors.

Within the TIME, TAMs showed their magnificent ability to promote cancer development. However, TAMs possess a robust tumorigenic ability that is associated with poor immune infiltration and survival in various cancers. Numerous studies have implicated the role of TAMs in enhancing several hallmarks of cancers such as tumor growth, vascularity, immune suppression, and therapy failure. Moreover, TAM heterogeneity and plasticity impact the TIME and its oncogenic functions to a greater extent. Recently, single-cell analysis revealed various TAM subsets within the TIME based on the molecular signatures and their functions. An illustration showing various TAM subsets is depicted in Figure 4.

**Figure 5:**
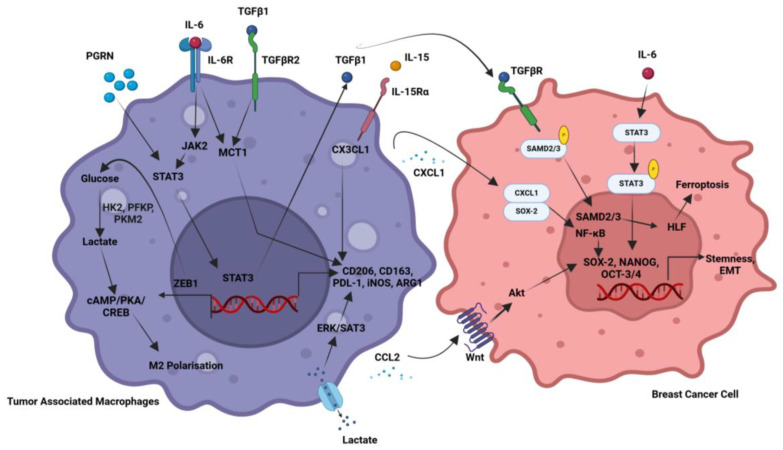
Illustration depicting molecular crosstalk between TAMs and cancer cells via various signaling pathways in promoting breast tumor development and metastasis.

For instance, PGRN upregulates PD-L1 expression and triggers M2 polarization via activation of STAT3, resulting in immunosuppressive PD-1/PD-L1 interaction in breast cancer [167]. TGF-β1/SMAD/HLF-activated IL-6 in TNBC cells induces a TAM-like phenotype via the JAK/STAT3 pathway and further upregulates TGF-β 1 expression, constituting a feedback circuit that promotes breast cancer ferroptosis resistance [168]. LncRNA-SNHG1 induces macrophage M2-like polarization, which leads to breast cancer growth and metastasis [169]. Transformed cells producing a high ectopic expression of ZEB1 generate lactate, which activates the PKA/CREB signaling cascade and results in the phenotype of alternatively activated (M2) macrophages in breast cancer [170]. Similarly, dysregulation of aerobic glycolysis, an increase in M2-like TAMs, and poor prognosis are clinically linked to Zeb1 expression in patients with breast cancer [170]. IL-15Rα+ve TAMs lowered CX3CL1 protein levels in tumor cells, inhibiting CD8+ve T cell recruitment via secretion of the IL-15/IL-15Rα complex (IL-15Rc). Thus, the IL-15Rc-HIF-1α-CX3CL1 signaling pathway links tumor cells with macrophages in the breast TME [171]. Moreover, TAMs induce immunotherapy resistance via secreting a rich array of cytokines and chemokines and restrict T cell infiltration in breast cancer [172]. Further, TAM-derived CCL2 triggers Ser-552 phosphorylation of β-catenin via Akt signaling, promotes EMT, and enriches cancer stemness [173]. TNBC cells expressing oncogenic multiple copies in T cell malignancy 1 (MCT-1) induces IL-6 via IL-6R and promotes macrophage polarization into M2-like macrophages while silencing MCT-1 and blocking IL-6R by tocilizumab, reducing IL-6R expression and macrophage polarization in breast cancer [174]. The heterogeneity of TAMs within the TIME is categorized based on the molecular signature and secretory factors. A recent study classified TAMs into pro-tumor and anti-tumor subsets based on the ratio of SPP1 and CXCL9 [175]. Consistent with this notion, another study showed that PD-L1+ve macrophages are immunostimulatory, while their absence creates an immunosuppressive TIME in breast cancer [176]. In addition, binding of TAM-derived CXCL1 to SOX4 promoter enhances its promoter activity via the NF-κB pathway, and silencing CXCL1 in TAMs showed a significant reduction in breast cancer progression and metastasis [177]. Similarly, CXCL1 expression is reduced by tumor cell-derived SPTBN1 and further inhibits macrophage polarization in breast cancer [178]. LYVE-1+ve TAMs are activated by IL-6, which causes them to express more immune-suppressive enzyme-like heme oxygenase-1, activates CCR5 signaling pathway, and controls the formation of nests. Additionally, LYVE-1+ve TAM formation or nest structure inhibition in gene-targeted mice improves CD8+ve T cell migration to the tumor and fosters a better response to treatment [179]. Tumor-derived lactate activates the ERK/STAT3 signaling pathway, which leads to M2 macrophage polarization in breast cancer [180]. miR-382 targets PGC-1α, reducing the TAM population with the M2 phenotype and potentially limiting breast cancer growth and metastasis [181]. EZH2 reduction resulted in DNA demethylation and subsequent elevation of miR-124-3p levels, inhibiting its target CCL2 production in tumor cells and inhibiting M2-type TAM polarization [182]. The immune-suppressive cytokine TGFβ1 and its related receptor TGFβR2 were increased in PMA-activated THP-1, which was stimulated by the CM of MDA-MB-231 cells, resulting in the cell surface expression of CD163. TNBC-induced TAMs exhibited higher expression of M1-associated genes such as CXCL10, IL-1β, and TNFα in comparison with THP-1-derived macrophages polarized by IL-4/IL-13 [183]. By improving the interaction between tumor cells and macrophages, the VEGFA/NRP-1/GAPVD1 axis facilitates the growth and cancer stemness of TNBC [184]. KLF14 inhibited the migration of breast cancer cells and the polarization of M2 macrophages via altering the signaling pathways of SOCS3/RhoA/Rock/STAT3. Further, M2 macrophage polarization was significantly reduced by either SOCS3 silencing or stimulation of RhoA/Rock/STAT3 signaling [185]. Recently, Xia et al. reported that TAM-derived IL-1β has been shown to interact with the transcription factor Yin Yang 1 (YY1), activate IL-1R2, and increase PD-L1 expression. This results in YY1 ubiquitination and proteasomal degradation via c-Fos activation, which in turn causes PD-L1 expression in both TAMs and TNBC cells. The combination of IL-1R2-neutralizing antibodies with anti-PD-1 resulted in increased anti-tumor efficacy while decreasing TAMs and exhausted CD8+ve T cells [186]. TAMs have a significant role in the immunological invasion of breast cancer by activating various signaling pathways as shown in Figure 5. TME signals cause functional reprogramming of recruited monocytes and tissue-resident macrophages, increasing cancer cell proliferation and metastasis while decreasing anti-tumor immunity. Further research is required to fully use the tumoricidal capability of macrophages, and data indicate that TAM reprogramming could complement immunotherapy in providing a robust anti-tumor response. Significant variability within the myeloid population was found by single-cell analysis, which also led to the identification of new lipid-associated macrophages (LAMs) that express PD-L1 and PD-L2, suggesting their immunoregulatory function [187]. These LAMs were found to predict poor clinical outcomes in large patient cohorts. In contrast to the macrophage polarization phenomenon, which suggests that M1 and M2 states are mutually exclusive and associated, genes were frequently expressed in the same cells. Thus, profiling with single-cell omics and spatial proteomics along with high-throughput sequencing of macrophage functional states and plasticity dictating tumorigenic ability could unravel novel therapeutic targets for the treatment modality in breast cancer.

## 6. Therapy Failure and Resistance

Breast cancer management is associated with a multidisciplinary approach that includes radiation therapy, chemotherapy, hormone treatment, targeted therapy, and surgery. Clonal evolution and various mutational loads with the cancer cell results in the acquiring of therapeutic resistance in clinical settings. Resistance mechanisms are achieved in two ways: (i) tumors may be intrinsically resistant prior to chemotherapy, or (ii) tumors that were previously responsive to chemotherapy can acquire resistance during treatment. Further, these resistance mechanisms, developed by various signaling cascades such as the PI3K/Akt/mTOR and RAS/MAPK/ERK pathways, can be activated in response to upstream signaling, contributing to chemoresistance [188]. A recent study showed that overexpression of Rac1 is linked with chemoresistance against multiple drugs used in neoadjuvant chemotherapy. Rac1 promotes resistance by activating the aldolase A and ERK signaling pathways, which enhance glycolysis and upregulate non-oxidative pentose phosphate pathways. This results in elevated nucleotide metabolism, helping breast cancer cells to withstand DNA damage caused by chemotherapy [189]. Liang et al. suggested that interaction of HSPB1 and IkB-α triggers HSPB1 ubiquitination-mediated degradation, which results in NF-κB signaling activation and nuclear translocation, contributing to doxorubicin (DOX) resistance. Additionally, higher levels of HSPB1 resulted in increased IL-6 secretion, further promoting the advancement of breast cancer [190]. In multidrug-resistant MCF-7 and cisplatin-resistant MDA-MB-468 cells, Akt phosphorylation, regulated by GSK3β and PTEN, is associated with cell viability, migration, and apoptosis, potentially contributing to chemoresistance in breast cancer. Moreover, GSK3β can affect cell viability through the PTEN/PI3K/Akt signaling pathway, thereby inducing chemoresistance [191]. Further investigation showed that the release of exosomes containing miR-378a-3p and miR-378d is induced by activation of the EZH2/STAT3 axis in breast cancer. Chemotherapy-resistant breast cancer cells subsequently take up these exosomes and activate the Wnt and Notch signaling in stem cells by selectively targeting DKK3 and NUMB, which ultimately leads to drug resistance [192]. Similarly, Pygo2, a co-activator of the Wnt/β-catenin pathway, was identified as the most expressed gene in resistant breast cancer cells. Subsequent studies demonstrated that Pygo2 activates the expression of MDR1 in resistant cells via Wnt/β-catenin signaling while silencing Pygo2 expression, which restored sensitivity to chemotherapy and decreased the BCSC population in response to treatment [193]. Follistatin-like 1 (FSTL1) expression is increased in MDA-MB-468 cells as well as doxorubicin-resistant MDA-MB-231 cells. However, luciferase assays indicated that miR-137 decreases FSTL1 at mRNA and protein levels. Consequently, it was demonstrated that the miR-137/FSTL1/integrinβ3/Wnt/β-catenin signaling cascade participates in the regulation of stemness and chemoresistance in breast cancer [194].

Jalalirad et al. suggested that AURKA is crucial for the TGF-β-mediated expression of SNAl1 [195]. Upregulation of SNAl1 leads to the accumulation of ALDH1 ^high^ breast tumor-initiating cells, thereby promoting chemoresistance in TNBC. Targeting both TGF-β and AURKA improved the sensitivity of patient-derived, chemoresistant TNBC cells to docetaxel-based treatment and reversed malignant plasticity [195]. Moreover, TGF-β1-enriched conditioned media generated using parental breast cancer cells that are triple-negative and estrogen receptor-positive, as well as their resistant counterparts, activate the p44/42 MAPK signaling pathway, which can enhance drug resistance and promote the CAF phenotype in dermal fibroblast. Notably, resistant breast cancer cells exhibited a greater capacity for fibroblast activation and showed an enhanced expression of mesenchymal markers as compared to parental cells in response to TGF-β1 [196]. NRP-1 has a crucial role in predicting the cellular response to drug treatment by inversely regulating ABCG2 expression. Overexpression of NRP-1 in BT-474 cells with lower ABCG2 expression sensitize these cells to adriamycin/cyclophosphamide thereby downregulating the NRP-1/ITGB3/FAK/Akt signaling. In contrast, wild-type BT-474 cells exhibit a lower expression of NRP-1 and higher ABCG2 expression, and administration of adriamycin/cyclophosphamide upregulates the NRP-1/ITGB3 cascade, thereby promoting chemoresistance [197]. Tamoxifen-resistant breast cancer cells exhibit significantly elevated levels of BARD1 and BRCA1, contributing to their resistance to DNA-damaging chemotherapy. Expression of BARD1 and BRCA1 are upregulated by activation of the PI3K/Akt pathway in these resistant breast cancer cells. Additionally, within both in vitro and in vivo models, PI3K inhibitors decrease the expression of BARD1 and BRCA1 in cells resistant to tamoxifen and re-sensitize them to cisplatin [198]. Another study uncovered that the expression of SerGlycin (SRGN) is elevated in chemoresistant breast cancer cells. The ITGA5/FAK/CREB/YAP signaling pathway is activated by extracellular SRGN, creating a positive feedback loop that is reliant on TEAD1. This mechanism induces HDAC2 expression, which helps the breast cancer cells to maintain stemness and promote chemoresistance [199]. Therefore, exploring novel resistant mechanisms induced by current therapeutics and their molecular drivers will unravel new avenues in breast cancer treatment strategies.

## 7. Epigenetic-Mediated Signaling

Breast cancer incidences are attributed to genetic factors, primarily involving genes like TP53, BRCA1, and BRCA2, which regulate genomic stability through cell cycle control, DNA repair, and apoptosis [200]. Apart from genetic mutations, the development of breast cancer is strongly influenced by epigenetic modifications such as histone modification and DNA methylation [201]. These epigenetic modifications exaggerate the development of breast tumors by silencing tumor suppressor genes or activating oncogenes. The reversibility of epigenetic alterations holds promising opportunities for therapeutic development. For instance, DNA methyltransferase (DNMT) and HDAC inhibitors are being investigated to restore normal gene expression patterns [202].

DNA methylation is a crucial epigenetic process that contributes significantly to maintain genomic integrity and regulate gene expression. Numerous malignancies are linked to aberrant DNA methylation, which is the covalent addition of a methyl group to CpG dinucleotides by the action of DNMT [203]. This can result in whole-genome hypomethylation in the genomic repeat regions and local hypermethylation of a gene’s promoter region in the CpG island [204]. Malignant neoplasms have been linked to both these changes. Moreover, particular hypomethylation of many genes, including signal-induced proliferation-associated 1 (SIPA1), has also been connected to the development of cancer [205].

The estrogen receptor plays an integral role in the epigenetic changes that lead to breast cancer; in particular, estradiol (E2) initiates breast cancer. E2 treatment stimulates breast cancer growth through proliferation and invasion [206]. One crucial epigenetic mechanism involves the polycomb group protein EZH2. EZH2 interacts with both ER and β-catenin, linking the estrogen and Wnt signaling pathways [207]. Wnt signaling plays a pivotal role in breast cancer development and progression. Antagonistic genes of Wnt signaling, such as SERP and DKK, are often silenced through epigenetic mechanisms like DNA methylation. This silencing leads to the continuous activation of β-catenin, promoting stem cell proliferation and contributing to poor prognosis [208,209].

## 8. Exosomal CircRNA-, miRNA-, and lncRNA-Mediated Signaling in Breast Cancer

Based on the ENCODE project, the majority of the human genome encodes numerous RNAs that lack translational ability [210]. Noncoding RNAs, commonly referred to as ncRNAs, are a family of RNAs that have garnered notable interest in the past few years for their critical gene regulatory functions, such as their involvement in multiple physiological activities, including apoptosis, cell cycle regulation, proliferation, migration leading to cancer progression [211]. Moreover, the advancement and metastasis of tumors are frequently linked to the deregulated expression of ncRNAs. Recent studies suggest that nc RNAs such as exosomal circular RNAs, long noncoding RNAs (lncRNAs), and miRNAs play a pivotal role in the pathogenesis and progression of breast cancer [211,212]. ncRNAs offer a potential avenue for cancer regulation through the identification of novel drug candidates. Moreover, these RNAs harbor unique properties that make them effective biomarkers for monitoring chemoresistance or recurrence in cancer management, including in breast cancer [213,214].

### 8.1. Exosomal Circular RNAs in Breast Cancer Pathogenesis

Exosomal circular RNAs play a pivotal role in the regulation of tumor proliferation, growth, and angiogenesis. Liang and his colleagues, based on a circRNA microarray, have reported several aberrantly expressed circRNAs, including circ-ABCB10 in breast cancer cells [215]. In addition, they also demonstrated that downregulation of circ-ABCB10 promotes G0/G1 arrest in breast cancer cells, followed by reduced clonogenicity and in vitro cellular proliferation [215]. The importance of exosomal circRNAs in metastasis and EMT has been shown previously by altering the tumor suppressor genes expression and sponging miRNAs. Studies conducted in vivo and in vitro indicate that circANKS1B promotes invasion and metastasis by triggering EMT via the TGF-β1 signaling pathway [216]. Further, a prior study demonstrated that circBCBM1 promotes the migration and progression of breast cancer. At the molecular level, circBCBM1 acts as a sponge on miR125a and influences BRD4 regulation, which results in alterations of MMP 9 expression through the SHH signaling pathway [217]. The inhibition of apoptosis and modulation of cancer-related gene expression is regulated through the circRNA/PI3K/Akt axis. Xu et al. have shown that hsa_circ_001569 was significantly upregulated in breast tumor tissues as well as cell lines [218]. As a consequence of hsa_circ_001569 silencing in these cell lines, it induced apoptosis by blocking the PI3K/Akt signaling activation, in addition to suppressing invasion and migration, as well altering the expressions of EMT markers [218].

Moreover, miR-148b-3p directly inhibits PTEN, an established tumor suppressor that causes tumor progression in breast cancer via negatively modulating the PI3K/Akt pathway [219]. Furthermore, Wang et al. have reported that the expression of circRNA_000911 is downregulated in breast cancer cells [220]. The apoptotic propensity was enhanced by sponging miR-449a and overexpressing circRNA_000911 in breast cancer cells. As a result, proliferation, invasion, and migration were reduced, thereby downregulating the Notch1 and NF-κB signaling pathways [220].

### 8.2. Oncogenic and Tumor Suppressor miRNAs in Breast Cancer Progression

Notably, miRNAs have been associated with various stages of breast cancer’s metastatic progression [221]. Breast cancer develops when abnormal expression of G2/S phase-regulatory proteins including EGFR and Akt signaling causes uncontrolled cell division. Many miRNAs, like miR-21, that are overexpressed in HER2+ve and triple-negative breast malignancies, target key modulators, such as PTEN, and promote tumor growth by suppressing PTEN and increasing PI3K/Akt signaling [222]. In addition to controlling PTEN expression, other miRNAs such as miRNA-93 and miRNA-106b also contribute to the aberrant activation of the PI3K/Akt pathway in breast cancer cells [223,224]. Furthermore, miR-424-5p increases PI3K and Akt activity and targets PTEN to foster the proliferation and invasion of breast cancer cells. The 3′UTR of PD-L1 mRNA can be precisely targeted by miR-424-5p, which leads to a decrease in mRNA as well as protein levels of PD-L1, thereby regulating PD-L1-driven PTEN/PI3K/Akt/mTOR pathway [225]. This observation provides additional evidence for the miRNA-driven regulation of PTEN expression. One possible therapeutic approach for breast cancer could involve targeting these miRNAs or upregulating PTEN. miR-99a attenuates mTOR signaling resulting in inhibition of breast tumor growth through the HIF1-α pathway [226].

Interestingly, miR34 family regulates breast cancer cell proliferation and is also associated with multidrug resistance in breast cancer, suggesting its dual role as a tumor suppressor and provider of oncogenic function [227]. miR-147 downregulation in breast cancer promotes Akt/mTOR signaling, while miR-200c enhances breast tumor cell sensitivity to doxorubicin and inhibits EMT and metastasis [228,229,230]. miR-204-5p upregulation inhibits breast cancer cell proliferation and metastasis by suppressing PI3K/Akt signaling through targeting PIK3CB [231].

### 8.3. Role of lncRNA-Mediated Signaling Pathways in Breast Cancer Development

LncRNAs, around 200 nucleotides in length, have currently been reported to harbor a significant role in the initiation and spread of breast cancer, as demonstrated by the latest studies. Extensive research has focused on investigating HOTAIR in breast cancer, a lncRNA that regulates the HOXC gene cluster and influences gene expression in various biological processes, including cancer progression [232]. Higher levels of HOTAIR expression in breast cancer tissues are associated with more aggressive tumor behavior and a poorer prognosis [233,234]. HOTAIR promotes invasion, tumor growth, and metastasis through multiple pathways, including the PI3K/Akt/mTOR signaling pathway, which is crucial for cellular metabolism [234]. Furthermore, recent investigations have demonstrated the importance of lncRNAs in regulating many signaling pathways, such as TGF-β, NF-κB, and Hedgehog, all of which have been associated with breast cancer [235,236]. Akt/PI3K/mTOR signaling cascade in breast cancer is intricately linked to other lncRNAs, such as MALAT1 and UCA1 [211,237]. Another crucial signaling cascade linked to the emergence of breast cancer is the Wnt/β-catenin pathway. LncRNAs like GAS5 and HOTAIR are thought to have an impact on the Hedgehog signaling pathway, whereas MALAT1 and HOTAIR are known to affect the TGF-β signaling pathway [238]. In addition, NF-κB signaling is influenced by lncRNAs such as HOTAIR and MEG3 in breast cancer [235].

## 9. Therapeutic Hotspots for Breast Cancer Treatment

**Figure 6:**
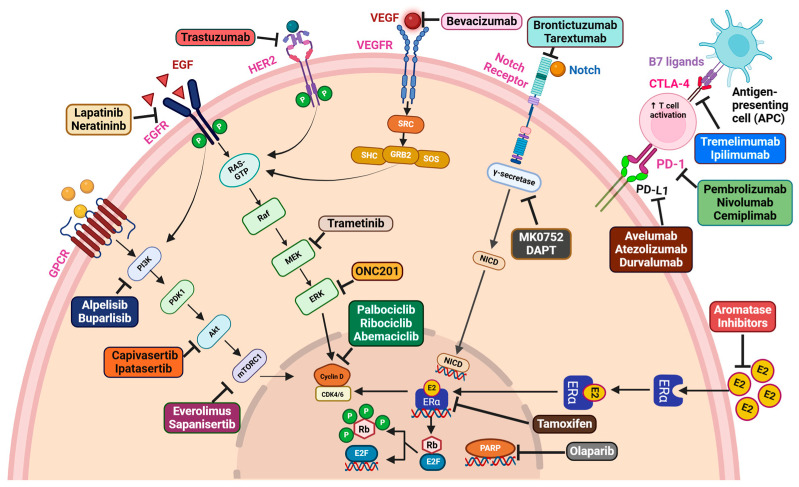
Representative molecular targets in breast cancer, along with the corresponding immunotherapeutic and targeted treatment approach. The GPCR-mediated AKT/PI3K/mTOR signaling pathway is a major target for inhibitors such as alpelisib, buparlisib, capivasertib, ipatasertib, everolimus, and sapanisertib, used to suppress breast tumor growth. Inhibitors including trametinib and ONC201 target the EGFR-activated MEK/ERK signaling pathway. Targeting HER2 and VEGF with their respective monoclonal antibodies, trastuzumab and bevacizumab, leads to the inhibition of cell proliferation and angiogenesis. Tarextumab, a potent inhibitor of the Notch receptor, along with MK0752 and DAPT, that target γ-secretase, inactivate the Notch signaling pathway. Palbociclib, ribociclib, and abemaciclib are the CDK4/6 inhibitors that induce cell cycle arrest by suppression of CDK4/6-mediated signaling. Targeting ER with hormone therapy like tamoxifen and aromatase inhibitors can be a key strategy for treating hormone receptor-positive breast cancer. Immune checkpoint inhibitors such as pembrolizumab, nivolumab, cemiplimab, avelumab, atezolizumab, durvalumab, tremelimumab, and ipilimumab block PD-1, PD-L1, and CTLA-4, respectively, act as effective immunotherapeutic drugs.

The conventional treatment strategy for breast cancer management includes radiation, surgery, and chemotherapy. Some of the major chemotherapeutic drugs and druggable molecules with their therapeutic targets are highlighted in Figure 6. Aromatase inhibitors, endocrine therapies, selective estrogen receptor degraders (SERD) and selective estrogen receptor modulators (SERM) are established approaches for personalized therapies in HR+ve breast cancer [239]. Tamoxifen was the first drug approved as a first line therapy in advanced breast cancer with higher ER status and reduces tumor recurrence [240]. Inhibitors that target PARP, HER2, PI3K, Akt, mTOR, EGF/EGFR, VEGF/VEGFR, and Notch could potentially be effective treatments to reduce the progression of breast cancer, as these pathways play key roles in various mechanisms of cancer development. Olaparib, a recognized PARP inhibitor, demonstrated greater efficacy than standard treatment in the phase III OlympiAD trial (NCT02000622). This trial conducted with olaparib alone resulted in a 2.8-month increase in PFS and reduced mortality in metastatic HER2− breast cancer patients with BRCA mutations [33]. In a phase III trial, olaparib showed an increased invasive DFS in patients with HER2-ve and gBRCA− pathogenic or likely pathogenic breast cancer (NCT02032823) [241].

One of the therapeutic hotspots gaining significant attention is the PI3K/Akt/mTOR pathway, which is known to be involved in promoting various oncogenic functions. The FDA approved alpelisib (BYL719), an p110α-specific PI3K inhibitor, as a therapeutic option for metastatic HR+ve and HER2-ve postmenopausal women [30]. Buparlisib (BKM120) is an oral pan-class I PI3K inhibitor that is under clinical trials in TNBC patients [242]. Patients with metastatic TNBC had a higher PFS rate when ipatasertib was combined with paclitaxel in the phase II LOTUS study (NCT02162719) [243]. Capivasertib, an ATP-competitive Akt inhibitor, significantly downregulates Akt signaling, as well as reduces breast tumor growth [244]. ONC201 belongs to a novel class of anticancer drugs called imipridones which is known to be a p53-independent transcriptional inducer of TNF-associated apoptosis-promoting ligand (TRAIL) [245,246,247]. ONC201 is linked to dephosphorylation of the FOXO3a and promotes the TRAIL gene expression by inactivating Akt and Erk [247]. Preclinical investigations demonstrated that ONC201 is effective in TNBC as well as non-TNBC cells that include BRCA1-deficient cells, irrespective of TRAIL sensitivity [248]. Trametinib, an MEK-specific inhibitor, has been shown to inhibit breast cancer growth [249]. Trametinib in combination with SHP099, an allosteric small-molecule inhibitor of SHP2, showed good efficacy in TNBC cells [250]. It exhibited synergistic effects across various cell lines, impeding reactivation of ERK in response to MEK inhibitors and hindering transcriptional activation [250].

Additionally, everolimus, an mTORC1 allosteric inhibitor, in conjunction with exemestane, was FDA-approved for the treatment of HR+ve and HER2-ve breast cancer. This trial including patients with ER+ve breast cancer showed that neoadjuvant letrozole treatment combined with everolimus prior to surgery had an improved clinical outcome and reduced tumor growth in contrast to letrozole treated alone [251]. Sapanisertib is a potent mTOR kinase inhibitor that selectively targets both mTORC1 and mTORC2. In a phase II trial, sapanisertib in combination with exemestane or fulvestrant exhibited therapeutic benefits in postmenopausal women with pretreated everolimus-resistant or sensitive breast cancer [252,253]. Furthermore, lapatinib and neratinib are the FDA-approved EGFR-TKIs for the treatment of HER2+ve breast cancer [254]. CDK4/6 is a crucial player in driving breast cancer progression. For targeting CDK4/6, the FDA has approved inhibitors such as palbociclib, abemaciclib, and ribociclib for the treatment of different forms of breast cancer [255]. Angiogenesis inhibitors can be applied as therapeutics to address different types of solid tumors, including breast. These inhibitors consist of small-molecule TKIs and monoclonal antibodies, primarily targeting VEGF and its receptors. In addition to bevacizumab, an anti-VEGF monoclonal antibody, paclitaxel chemotherapy increased the PFS in patients with metastatic breast cancer (NCT00028990) [256].

The breast cancer TME is generally considered as a “cold TIME”, suggesting a highly non-immunogenic condition. Various approaches to regulate the immune system have been explored in clinical trials. In addition to the recognized breast cancer subtypes, the identification of prognostic biomarkers is important for customizing immunotherapies. According to the phase II I-SPY2 trial, patients with HR+ve and HER2-ve breast cancer or TNBC showed a significantly higher pathologic response when cemiplimab, a PD-1 targeting IgG4 monoclonal antibody and fianlimab, a LAG-3 inhibitor were combined with paclitaxel (NCT01042379) [257]. Combining the PARP inhibitor olaparib with durvalumab showed promising anti-tumor activity in an open-label phase I/II trial involving patients with metastatic breast cancer who have a gBRCA mutation (NCT02734004) [258]. Ipilimumab is an IgG1 monoclonal antibody that targets CTLA-4 and exhibits substantial anti-tumor efficacy across a range of cancers. According to preclinical data, ipilimumab induces the secretion of IL-2 by TNBC cells into the TME, thereby strengthening the immunological response [259]. In phase II clinical trials, the combination therapy of ipilimumab and nivolumab along with neoadjuvant paclitaxel showed promising pathological responses in early-stage TNBC patients [260]. Tremelimumab is a humanized IgG2 monoclonal antibody that targets CTLA-4 thereby preventing tumor growth by blocking CTLA-4 and B7 interactions [261].

Anticancer vaccines are designed to stimulate antigen-specific T cell activation to target cancer cells. Breast cancer vaccines can be broadly categorized into two types. One of these targets HER2 or HER2-associated antigens and the other focuses on non-HER2-related antigens. The effectiveness of two HER2 peptide vaccines, GP2 and AE37, has been compared in a single-blind phase II trial in patients with HER2+ve breast cancer [262]. While GP2 immunization resulted in recurrence-free survival to HER2-overexpressing subgroups, AE37 vaccination conferred therapeutic benefit in TNBC and patients representing low HER2 [263]. A recombinant fowl pox vector encoding tumor-associated antigens such as carcinoembryonic antigen (CEA) and MUC1 is part of the PANVAC recombinant viral vaccine, which also comprises a second viral vector containing transgenes for cancer-associated antigens and the co-stimulatory drug TRICOM [264]. Patients with metastatic breast cancer undergoing docetaxel therapy with PANVAC demonstrated increased PFS compared to the control group in a phase II trial (NCT00179309) [264].

An increasing number of clinical trials are being conducted to develop effective breast cancer therapy, as depicted in Table 2. However, achieving the intended treatment outcome with targeted or immune therapy requires a thorough understanding of the genetic, molecular, and immunological landscape of tumor cells as well as the TME.

## 10. Conclusions

Breast cancer is one of the most prevailing female malignancies around the globe. Its intricate microenvironmental landscape demands further comprehensive investigation. The breast TME unveils a labyrinthine network system comprising multilevel intercellular interactions among the immune cells, stromal cells, and other cellular components. These interactions are operated by an array of molecular signaling pathways and unravel a rich tapestry of avenues for targeting a myriad of novel molecular players in finding effective therapeutic modalities. Many aberrations and genetic interplays among breast cancer subtypes are yet unknown in terms of function. To deconvolute critical molecular pathways and interconnecting nodes, rigorous functional screening (for example, utilizing CRISPR-Cas9 editing or spatial omics studies) as well as deeper analysis and data integration are warranted.

Numerous studies have dissected multiple signaling pathways and the molecules involved in aiding breast tumor growth and metastasis. For instance, our group has reported many signaling pathways regulating various oncogenic functions within the breast TME and multiple therapeutic options by targeting those molecular players [262]. One of the studies reported that tumor-derived OPN activates Twist1-dependent gene expression by binding to CD44 and αvβ3 integrins on the CAF via Akt and ERK pathway, causing tumor cells to undergo EMT [265]. In addition, Radharani et al. showed that breast tumor-activated macrophages enrich cancer stemness by upregulating CSC specific transcription factors via activating the IL-6/STAT3 pathway [266]. Recently, Panda et al. reviewed extensively OPN-regulated pathways and their downstream implications in breast and other cancers [267]. Furthermore, curcumin–chitosan nanoparticles (Cur-CHNPs), either alone or in combination with 5-FU, reduced the expression of OPN and VEGF by dysregulating PI3K and Akt activation, thereby inhibiting breast cancer migration and metastasis [268].

This review comprehensively gleaned the various fundamental signaling pathways orchestrated by numerous cellular and molecular players, focusing on the pan-breast TME. Further, this review summarized the existing in-depth analysis of current breast cancer therapies, as well as stromal- and immune-cell mediated oncogenic signaling pathways that enhance several hallmarks of breast cancer. Moreover, the multifaceted classification of breast tumors and novel therapeutic regimens have been comprehensively discussed. The array of molecular signaling regulating EMT dictates breast tumor growth and metastasis. These regulated networks within the breast TME promote tumor angiogenesis, specifically VEGF-dependent and independent pathways, CSC enrichment, tumor metabolism, therapy failure, and autophagy. In addition, the role of oncogenic pathways in the breast TME has been explored thoroughly, focusing on stromal cells such as CAFs, adipocytes, and mast cells, and immune cells such as macrophage-mediated regulatory networks resulting in breast tumor growth and metastasis. Furthermore, it has been addressed that noncoding RNAs, such as exosomal circular RNAs, lncRNAs, and miRNAs, are crucial for the initiation and spread of breast cancer. Therefore, targeting these noncoding RNAs could be a better therapeutic option for the management of breast cancer. An in-depth exploration of therapeutic modalities and their potential clinical utilities impacting numerous microenvironmental cellular and molecular components within the breast TME have been discussed.

## 11. Future Perspective

Although we have highlighted several regulated networks and pathways as well as their implications in breast cancer, a deeper understanding of the microenvironmental landscape is required. Furthermore, in response to these issues, we propose numerous future research challenges: First, delving into the microenvironmental cues and developing effective mono- or combination therapeutic regimens. Second, in-depth investigation of cellular and molecular players as robust biomarkers for the diagnosis of breast cancer. Third, investigating precise therapeutic options for microenvironment modulation, including targeted therapy for specific biomarkers and immunomodulatory therapy, to enhance tailored treatment and effectiveness. Exploring these future research avenues will enhance our understanding of the breast microenvironment and boost the clinical utility of microenvironmental biomarkers among molecular players, which could open new dimensions in breast cancer therapy.

## Figures and Tables

**Table 1:** Recent FDA-approved drugs for breast cancer (source: https://www.fda.gov/) (accessed on 27 December 2024).

Drug Name	Active Ingredient	Breast Cancer Type
KISQALI KISQALI FEMERA CO-PACK (CO-PACKAGE)	RIBOCICLIB SUCCINATE LETROZOLE; RIBOCICLIB SUCCINATE	HR+ve, HER2-ve breast cancer
ENHERTU	FAM TRASTUZUMAB DERUXTECAN-NXKI	HER2+ve breast cancer
TEPYLUTE	THIOTEPA	Breast adenocarcinoma
IBRANCE tablets	PALBOCICLIB	HR+ve, HER2-ve, advanced, or metastatic breast cancer
HALAVEN-injection	ERIBULIN MESYLATE	Metastatic breast cancer
TRUQAP	CAPIVASERTIB	HR+ve, HER2-ve, locally advanced metastatic breast cancer
ORSERDU	ELACESTRANT HYDROCHLORIDE	ER+ve, HER2-ve, ESR1-mutated, advanced, or metastatic breast cancer
VERZENIO with endocrine therapy (tamoxifen or an aromatase inhibitor or fulvestrant	ABEMACICLIB	HR+ve, HER2-ve, node-positive early and advanced, or metastatic breast cancer
LYNPARZA	OLAPARIB	Germline BRCA-mutated, HER2-ve breast cancer
KEYTRUDA	PEMBROLIZUMAB	Triple-negative breast cancer
TRODELVY	SACITUZUMAB GOVITECAN	Metastatic triple-negative breast cancer

**Table 2:** Ongoing breast cancer clinical trials (Source: https://clinicaltrials.gov/) (accessed on 27 December 2024).

S. No.	Breast Cancer Subtype	Therapy Details	Phase	Clinical Trial No.
1	TNBC	Pembrolizumab, axatilimab, radiation therapy	Phase 2	NCT05491226
2	TNBC	Utidelone (UTD1) plus capecitabine	Phase 2	NCT06385990
3	TNBC	Carboplatin, docetaxel, doxorubicin, cyclophosphamide, pembrolizumab	Phase 2	NCT05645380
4	TNBC	Fluzoparib + paclitaxel Epirubicin + cyclophosphamide	Phase 2	NCT05834582
5	TNBC	Sintilimab, anlotinib, nab-paclitaxel, carboplatin, epirubicin, cyclophosphamide	Phase 2	NCT04877821
6	TNBC	Capecitabine	Phase 2	NCT04768426
7	TNBC	Anti-PD-1 monoclonal antibody, VEGFR2 tyrosine kinase inhibitor	Phase 2	NCT05556200
8	TNBC	Adebrelimab + stereotactic radiotherapy + nab-paclitaxel + carboplatin, adebrelimab + chemotherapy (nab-paclitaxel + carboplatin)	Phase 2	NCT06165900
9	TNBC	Epirubicin, cyclophosphamide, paclitaxel, carboplatin,	Phase 3	NCT03876886
10	TNBC	Deferoxamine plus chemotherapy	Phase 2	NCT05300958
11	TNBC	Tiragolumab, atezolizumab and chemotherapy	Phase 2	NCT06175390
12	TNBC	Epirubicin, CTX, paclitaxel, ddEpirubicin, ddCTX, paclitaxel (with carbo), carboplatin	Phase 3	NCT04296175
13	TNBC	BL-B01D1, eribulin, vinorelbine, gemcitabine, capecitabine	Phase 3	NCT06382142
14	TNBC	Olaparib, paclitaxel + carboplatin	Phase 2, 3	NCT03150576
15	TNBC	Sacituzumab govitecan-hziy (SG), Pembrolizumab, capecitabine	Phase 3	NCT05633654
16	TNBC	Albumin-bound paclitaxel + carboplatin, epirubicin + docetaxel	Phase 4	NCT04136782
17	TNBC	AZD6738, olaparib, durvalumab	Phase 2	NCT03740893
18	TNBC	Eribulin, LM-108, nab-paclitaxel, toripalimab	Phase 2	NCT06387628
19	TNBC	Tiragolumab, atezolizumab, ipilimumab	Phase 2	NCT06342037
20	TNBC	α-lactalbumin vaccine, zymosan	Phase 1	NCT04674306
21	TNBC	Ceralasertib, durvalumab, nab-paclitaxel	Phase 2	NCT05582538
22	TNBC	Capecitabine, talazoparib, pembrolizumab, inavolisib	Phase 2	NCT04849364
23	TNBC	Ribociclib, bicalutamide	Phase 1, 2	NCT03090165
24	TNBC	Camrelizumab plus famitinib with/without nab-palitaxel	Phase 2	NCT05670925
25	TNBC	Lenvatinib, pembrolizumab	Phase 1	NCT04427293
26	TNBC, stage IV breast cancer	Anti-HER2/HER3 dendritic cell vaccine, pembrolizumab	Phase 2	NCT04348747
27	TNBC (stage I, II, III breast cancer)	Carboplatin, cyclophosphamide, docetaxel, doxorubicin, paclitaxel, pembrolizumab	Phase 3	NCT05929768
28	Metastatic TNBC	Trilaciclib, pembrolizumab, gemcitabine, carboplatin	Phase 2	NCT06027268
29	Metastatic TNBC	Gemcitabine and carboplatin plus antibiotic (moxifloxacin); gemcitabine combined with carboplatin plus placebo	Phase 3	NCT04722978
30	TNBC, metastatic breast cancer	Sacituzumab govitecan	Phase 3	NCT05552001
31	TNBC, intermediate and high-risk luminal	Accelerated partial breast irradiation, chemotherapy	Phase 1, 2	NCT02806258
32	TNBC, ductal carcinoma in situ, lobular carcinoma in situ	Abemaciclib	Phase 2	NCT03979508
33	TNBC, HR+ve and HER2-ve breast cancer	Pembrolizumab, paclitaxel, carboplatin, cyclophosphamide, doxorubicin, capecitabine	Phase 2	NCT04443348
34	Invasive breast cancer (HR+ve, HER2-ve, or TNBC)	Cemiplimab, paclitaxel, carboplatin (not mandatory), doxorubicin, cyclophosphamide	Phase 2	NCT04243616
35	TNBC, stage III, IV, and Recurrent Breast Cancer	Avelumab, binimetinib, utomilumab, liposomal doxorubicin, sacituzumab govitecan	Phase 2	NCT03971409
36	Metastatic TNBC	Atezolizumab, bevacizumab, gemcitabine, carboplatin	Phase 2	NCT04739670
37	TNBC, metastatic breast cancer, HER2 -ve breast cancer	L-NMMA	Phase 2	NCT05660083
38	Metastatic TNBC, stage IV breast cancer	Ivermectin, balstilmab	Phase 1 & 2	NCT05318469
39	TNBC, metastatic breast cancer and ER-low breast cancer	Carboplatin, tocilizumab	Phase 2	NCT05846789
40	Breast cancers and metastatic AR+ve TNBC	Palbociclib, avelumab	Phase 1	NCT04360941
41	Unresectable or metastatic TNBC	Tobemstomig, pembrolizumab, nab-paclitaxel	Phase 2	NCT05852691
42	All types of breast cancer like HER2+ve and -ve and PR +ve, TNBC	ALX148, fam-trastuzumab deruxtecan-nxki, zanidatamab, tucatinib	Phase 1	NCT05868226
43	Breast cancer	Atezolizumab injection, bevacizumab, pertuzumab, trastuzumab	Phase 2	NCT05180006
44	Breast cancer	Nivolumab, ipilimumab	Phase 2	NCT03815890
45	HER2+ve, metastatic breast cancer	Inavolisib, Phesgo, taxane-based chemotherapy	Phase 3	NCT05894239
46	HER2+ve metastatic breast cancer	Atezolizumab + trastuzumab + vinorelbine	Phase 2	NCT04759248
47	Luminal breast cancer: HER2-ve HR+ve	Elacestrant, triptorelin	Phase 2	NCT05982093
48	Luminal A breast cancer	Breast irradiation (RT), endocrine therapy (ET): letrozole, anastrozole, exemestane, tamoxifen	Phase 3	NCT04134598
49	Luminal B/HER2-ve breast cancer	Dalpiciclib combined with aromatase inhibitors	Phase 2	NCT05640778
50	Ductal carcinoma in situ	Tamoxifen, exemestane, letrozole, anastrazole, testosterone + anastrazole, elacestrant, Z-endoxifen	Phase 2	NCT06075953
51	Ductal carcinoma in situ	Granulocyte–macrophage colony-stimulating factor, multi-epitope HER2 peptide vaccine H2NVAC	Phase 1	NCT04144023
52	Ductal carcinoma in situ, postmenopausal	Conjugated estrogens/bazedoxifene	Phase 2	NCT02694809
53	Invasive lobular carcinoma	Fulvestrant, repotrectinib	Phase 2	NCT06408168
54	Invasive breast lobular carcinoma	Neratinib	Phase 2	NCT05919108
55	HER2-ve breast cancer	Doxorubicin, cyclophosphamide Weekly paclitaxel, trastuzumab Pertuzumab	Phase 2	NCT03412643
56	ER+ve breast cancer, HER2-ve breast cancer, metastatic breast cancer	AI + CDK4/6i, SERD + CDK4/6i, mTOR inhibitor + AI, mTOR inhibitor + SERD, mTOR inhibitor + selective estrogen receptor modulator, PI3K inhibitor + SERD, PI3K inhibitor + AI, oral SERD	Phase 2	NCT05826964
57	HR+ve/HER2-ve metastatic breast cancer	Fulvestrant Capecitabine oral product	Phase 3	NCT04263298
58	HER2-low, HR+ve metastatic breast cancer	DB-1303/BNT323, capecitabine, paclitaxel, nab-paclitaxel	Phase 3	NCT06018337
59	ER+ve/HER2-ve metastatic breast cancer	Endocrine therapy combined with the local treatment of FES-negative lesions	Phase 3	NCT06195709
60	HER2-ve breast cancer	Cyclophosphamide, fludarabine, camrelizumab, chemotherapeutic drug, ADC, or PARP inhibitor	Phase 1	NCT06121557
61	HER2-ve breast cancer	Epirubicin, cyclophosphamide, docetaxel, paclitaxel	Phase 2 and 3	NCT04576143
62	HER2-ve breast cancer	Cyclophosphamide, Fludarabine, nab-paclitaxel, Gemcitabine, carboplatin	Phase 1 and 2	NCT05981001
63	HER2-ve breast cancer	Paclitaxel, carboplatin, Cyclophosphamide/doxorubicin	Phase 2 and 3	NCT05889390
64	HER2-ve breast cancer	Doxorubicin, epirubicin, cyclophosphamide, fludarabine Nab-paclitaxel	Phase 1	NCT06121570
65	ER+ve/HER2-ve breast cancer	Docetaxel, carboplatin, epirubicin, cyclophosphamide	Phase 3	NCT05901428
66	HR+ve/HER2-ve premenopausal breast cancer	Dalcelli, exemestane, gosserine Docetaxel, epirubicin hydrochloride, cyclophosphamide	Phase 2 and 3	NCT06009627
67	HER2-ve breast carcinoma, HR+ve	Cyclophosphamide, Doxorubicin, Durvalumab, paclitaxel	Phase 3	NCT06058377
68	HER2-ve early breast cancer	Liposomal doxorubicin, cyclophosphamide vs. docetaxel, cyclophosphamide	Phase 4	NCT05302336
69	HER2-ve breast cancer	Camrelizumab with vinorelbine and cisplatin	Phase 2	NCT04848454
70	HER2-ve breast carcinoma	Cyclophosphamide, Docetaxel	Phase 2	NCT06042569
